# Reduced Visual Cortex Gray Matter Volume and Thickness in Young Adults Who Witnessed Domestic Violence during Childhood

**DOI:** 10.1371/journal.pone.0052528

**Published:** 2012-12-26

**Authors:** Akemi Tomoda, Ann Polcari, Carl M. Anderson, Martin H. Teicher

**Affiliations:** 1 Department of Psychiatry, Harvard Medical School, Boston, Massachusetts, United States of America; 2 Developmental Biopsychiatry Research Program, McLean Hospital, Belmont, Massachusetts, United States of America; 3 Research Center for Child Mental Development, University of Fukui, Fukui, Japan; 4 School of Nursing, Northeastern University, Boston, Massachusetts, United States of America; 5 Brain Imaging Center, McLean Hospital, Belmont, Massachusetts, United States of America; University of Texas Southwestern Medical Center, United States of America

## Abstract

Exposure to interparental violence is associated with negative outcomes, such as depression, post-traumatic stress disorder and reduced cognitive abilities. However, little is known about the potential effects of witnessing domestic violence during childhood on gray matter volume (GMV) or cortical thickness. High-resolution 3.0 T volumetric scans (Siemens Trio Scanner) were obtained on 52 subjects (18–25 years) including 22 (6 males/16 females) with a history of visually witnessing episodes of domestic violence, and 30 (8 males/22 females) unexposed control subjects, with neither a current nor past DSM-IV Axis I or II disorder. Potential confounding effects of age, gender, level of parental verbal aggression, parental education, financial stress, full scale IQ, and total GMV, or average thickness were modeled using voxel based morphometry and FreeSurfer. Witnessing domestic violence subjects had a 6.1% GMV reduction in the right lingual gyrus (BA18) (*P* = 0.029, False Discovery Rate corrected peak level). Thickness in this region was also reduced, as was thickness in V2 bilaterally and left occipital pole. Theses regions were maximally sensitive to exposure to witnessing domestic violence between 11–13 years of age. Regional reductions in GMV and thickness were observed in both susceptible and resilient witnessing domestic violence subjects. Results in subjects witnessing domestic violence were similar to previously reported results in subjects with childhood sexual abuse, as the primary region affected was visual cortex. Brain regions that process and convey the adverse sensory input of the abuse may be specifically modified by this experience, particularly in subjects exposed to a single type of maltreatment. Exposure to multiple types of maltreatment is more commonly associated with morphological alterations in corticolimbic regions. These findings fit with preclinical studies showing that visual cortex is a highly plastic structure.

## Introduction

Witnessing domestic violence (WDV) is a highly stressful and potentially traumatic event. Approximately 15.5 million children in the U.S. witness DV annually [1]. Although many parents try to shelter their children from WDV, children in violent homes commonly see, hear, and intervene in episodes of WDV [2]. WDV increases risk for depression [3] and aggression [4], [5] by 2–4 fold, and is a frequent causes of childhood PTSD [6], [7].

Is WDV during childhood associated with enduring effects on brain morphometry? We recently reported a reduction in the integrity of the inferior longitudinal fasciculus (ILF) interconnecting visual cortex to limbic system in a sample of young adults who witnessed interparental violence during childhood [8]. The aim of this study was to investigate whether WDV during childhood was associated with enduring differences in GMV. Voxel based morphometry (VBM) was used to provide an unbiased, even-handed, whole-brain, voxel-by-voxel assessment in a healthy community sample. FreeSurfer, a software program for cortical surface-based reconstruction and analysis [9], [10], was then used in a more focused manner to verify and extend the findings.

## Methods

### Ethics Statement

This Project has been reviewed and approved by the McLean IRB, Assurance # 00002744. During the review of this Project, the IRB specifically considered (i) the risks and anticipated benefits, if any, to subjects; (ii) the selection of subjects; (iii) the procedures for securing and documenting informed consent; (iv) the safety of subjects; and (v) the privacy of subjects and confidentiality of the data. All participants gave written informed consent prior to participation.

### Participants

#### Recruitment and screening

Participants were recruited from the community using methods previously detailed [8], [11], [12], [13]. Our goal was to recruit unmedicated right-handed subjects 18–25 years of age who visually witnessed episodes of DV but were not exposed to childhood sexual abuse parental loss, neglect, physical maltreatment or other traumatic events. Subjects were also required to be free from neurological disease or insult, including head trauma resulting in loss of consciousness >5 minutes or migraine headaches. Subjects were excluded with a history of premature birth or birth complications; maternal substance abuse during pregnancy; or medical disorders that could affect brain development.

All potentially eligible subjects from online screenings were invited to the laboratory and underwent detailed evaluation, including Structural Clinical Interviews for DSM-IV Axis I and II psychiatric disorders (SCID) [14]. Exposure to interfamilial violence and other forms of maltreatment were assessed using the 100-item semi-structured Traumatic Antecedents Interview [15]. This interview was designed to evaluate reports of childhood sexual abuse, physical abuse, WDV, physical or emotional neglect, significant separations or losses, parental verbal abuse, or parental discord [16]. Certified mental health clinicians conducted the assessment and evaluation interviews. A panel of three doctoral-level psychiatric clinicians with extensive experience treating trauma-exposed individuals reviewed information on potential subjects and group assignments were made by full consensus. WDV subjects were selected without regard to psychiatric history, except for alcohol or drug abuse, which were exclusion factors. Selecting subjects meeting criteria for a specific disorder could bias results by only including the most severely affected subjects. Conversely, selecting subjects without any psychiatric history could bias results in the opposite direction. The intent was to recruit a balanced sample that would provide a rigorous test of our proposed hypotheses.

Subjects provided written informed consent prior to completing the online screening instrument, and again before interviews and brain imaging. The present sample overlaps with a previous reported sample used to study fiber tract integrity [8], but includes two more subjects with WDV and three additional controls.

#### Subjects

Twenty-two subjects (6 males, 16 females; mean age, 21.8±2.4 years) with a history of WDV and 30 healthy age-equivalent controls (8 males, 22 females; mean age, 21.6±2.1 years) who met full criteria were imaged. Subjects in the WDV group reported seeing and hearing years of intense verbal aggression between their parents, which culminated in some years in acts of physical violence. Overall, they reported that they witnessed 3.8±3.5 (mean ±S.D.) years of exposure to interparental physical plus verbal aggression (IP-PA) along with 6.0±4.8 years of exposure to interparental verbal aggression without physical violence (IP-VA), for a total duration of 9.8±3.2 years. Controls had no histories of exposure to abuse, traumatic events, or harsh corporal punishment, and did not meet criteria for any major Axis I or Axis II psychiatric disorder. All participants were right-handed and unmedicated.

### Assessments

#### Abuse and trauma ratings

Exposure to parental verbal abuse was assessed with the Verbal Aggression Scale [17], which provides a continuous measure of exposure. Self-report ratings of psychiatric symptoms were obtained using Kellner’s Symptom Questionnaire [18]. Ratings of dissociation and ‘limbic irritability’ were obtained using the Dissociative Experience Scale [19] and limbic system checklist-33 [20].

Childhood poverty may be an important risk factor for psychopathology and affect trajectories of brain development. Young adult subjects were often uncertain about parental income while they were growing up. However, they were well aware of the degree of *perceived financial sufficiency,* or stress they experienced during this time. This was rated with a Likert item ranging from 1 (much less than enough money for our needs) to 5 (much more than enough money for our needs). Perceived financial sufficiency explained a greater share of the variance in symptom ratings than combined family income.

#### MRI acquisition and analysis

Image analysis was performed on high-resolution, T1-weighted MRI datasets, which were acquired on a Trio Scanner (3T; Siemens AG, Siemens Medical Solutions, Erlangen, Germany). An inversion prepared 3D MPRAGE sequence was used with an eight-element phased-array RF reception coil (Siemens AG). GRAPPA acquisition and processing was used to reduce the scan time, with a GRAPPA factor of 2. Scan parameters were: the sagittal plane, TE/TR/TI/flip = 2.74 ms/2.1 s/1.1 s/12 deg; 3D matrix 256×256×128 on 256×256×170 mm field of view; bandwidth 48.6 kHz; scan time 4:56.

VBM using Statistical Parametric Mapping (SPM8; Wellcome Department of Imaging Neuroscience, University College, London) was conducted as previously described [11], [21], [22]. Briefly, coarsely segmented images were registered to a standard template [23], [24] that conforms to the space defined by the ICBM, NIH P-20 project. Volume changes induced by normalization were adjusted via a modulation algorithm. Spatially normalized images were segmented into gray and white matter and smoothed using a 12-mm full-width half-maximum isotropic Gaussian kernel to generate a statistical parametric map. Between-group differences were analyzed using a general linear model. Potential confounding effects of age, sex, parental education, perceived financial sufficiency, parental verbal abuse, full scale IQ and whole segment GMV were modeled, and attributable variances excluded. Statistical threshold was set at *P*<0.05 with correction for multiple comparisons at cluster level (height threshold of *Z*>3.09) because of the increased sensitivity of clusters to detect spatially extended signal changes [25], [26]. Inference testing was based on the theory of Gaussian fields [27]. Potential problems relating to non-isotropic smoothness, which can invalidate cluster level comparisons [23], were corrected by adjusting cluster size from the resel per voxel image [25], [28].

VBM is a potentially powerful technique but it hinges on a number of assumptions, particularly the accuracy of image co-registration [29]. Hence, VBM findings were reevaluated using an independent technique that does not rely on co-registration. Cortical surface-based analysis was performed using FreeSurfer (version 5.1; Laboratory for Computational Neuroimaging, Martinos Center for Biomedical Imaging, Boston, MA) [9], [10], [30]. Each subject’s reconstructed brain was converted to an average spherical surface representation that optimally aligned sulcal and gyral features for the individual subject [9], [10]. Subdivision of the cortical ribbon into gyral-based subdivisions resulted in the identification of 82 validated cortical parcellation units per hemisphere. By application of the original deformation algorithms in reverse, ROIs were mapped back on to each unfolded surface [10]. Differences between WDV and control groups in thickness were assessed using analysis of covariance (ANCOVA) with parental education, financial sufficiency, age, gender degree of exposure to PVA, and mean cortical thickness as covariates. Parcellation regions selected for analysis were located in and around the areas of greatest difference identified by VBM. They included visual areas V1, V2 and V5/MT, lingual, fusiform, superior, middle and inferior occipital gyri, occipital pole and cuneus. Correction for multiple comparisons were made using the False discovery rate (FDR) method of Benjamini and Hochberg [31].

#### Statistical analyses

Data analyses were conducted using R [32]. Differences between groups were evaluated using ANCOVA. The primary hypothesis tested was whether there were significant regional brain differences between subjects who witnessed DV and unexposed psychiatrically healthy controls.

#### Susceptible versus resilient subjects

Four additional analyses were performed to extend these findings. In the first we divided the WDV group into susceptible subjects who met criteria for either major depression, PTSD, anxiety disorders, eating disorders or personality disorders following exposure to interparental violence (n = 13) and relatively resilient subjects who have not met criteria for any of these disorders (n = 9). This division was used to determine whether observed group differences in volume or thickness were specific to the susceptible subgroup.

#### Witnessing interparental verbal aggression versus physical aggression

Second, we assessed whether witnessing interparental physical aggression (IP-PA) was more strongly associated with alterations in regional GMV or thickness then witnessing episodes of interparental verbal aggression (IP-VA). The relative statistical impact of duration of exposure to IP-VA versus IP-PA on measures of thickness or volume (adjusted for age, gender and total GMV or mean cortical thickness) was determined using multiple regression. Additional regressors included parental education, perceived financial sufficiency and exposure to parental verbal abuse. The variance decomposition method of Lindeman et al., [33], [34] was used to more accurately determine the proportion of the variance attributable to each regressor, as these regressors were not entirely independent (R package *relaimpo*).

#### Sensitive period analysis

Third, the presence of a potential ‘sensitive period’ when childhood exposure to WDV was most strongly associate with adult measures of volume or thickness was assessed using random forest regression with conditional inference trees (‘*cforest* ‘in R package *party* [35]). This is a new computationally intensive analytic procedure based on decision trees. It is a form of “ensemble learning” in which a large number of unpruned decision trees are generated and their results aggregated. Advantages of random forest regression include: (1) very high classification accuracy; (2) a novel method of determining variable importance; (3) no restrictions regarding the distribution and scaling properties of the data; and (4) high tolerance for multicolinearity [36], [37]. We used a variant of Breiman's approach with conditional trees as the base learners to avoid a potential problem with biased estimates that can emerge when variables differ in range or number of categories [35]. For these analyses 1000 trees were generated with four variables randomly selected for evaluation at each node. Conditional forest regression indicates importance by assessing the decrease in accuracy of the forest's fit following permutation (effective elimination) of a given predictor variable. The more the permutation of a variable decreases accuracy the greater the importance of the variable. Cross-correlations in exposure rates between different ages were also controlled [35]. Exposure to interparental violence from ages 3–16 was coded with 0 for no exposure, 1 for witnessing IP-VA and 2 for witnessing IP-PA in a given year. Conditional forest regression determined the relative importance of exposure at each age on measures of volume or thickness adjusted for age, gender, perceived financial sufficiency, parental education, PVA and total GMV or mean cortical thickness.

#### Symptom ratings and brain measures

Finally, exploratory correlation analyses assessed whether differences in GMV in the area that differed most significantly between WDV subjects and controls could potentially account for a significant portion of the variance in pre-specified symptom ratings. Both cases and controls were included in the analyses, as we were interested in assessing potential functional correlates of these GMV differences in the general population, not just in subjects with WDV. Examining correlations in only one group, as sometimes advocated, restricts the range of the independent variable and can seriously bias results. Range restrictions deflate correlation coefficients if they reduce the standard deviation of the distribution of scores on one or both variables, and inflate r values if they increase the standard deviation, and should be avoided when possible [38]. Further, the size of the full sample provided sufficient power (0.8) to detect medium size effects (r∼0.4). Analysis of the individuals groups only provided sufficient power (0.8) to reliably detect large effects. However, within group analyses were examined for variables that showed a significant relationship with GMV in the entire subject pool to determine if the regressive relationship applied to one group more than the other. Partial correlation analysis was used to minimize the influence of age and gender on the strength of the association.

## Results

### Demographics and IQ

The two groups were well matched in gender, age, and education (Table 1). There was about a two-year difference in extent of parental education, and significant differences in perceived financial sufficiency and exposure to parental verbal abuse. WDV subjects indicated that their family’s financial resources were on average nearly adequate, while controls indicated that they were more than adequate. Subjects in these two groups also had similar mean performance scores on memory tests. There were no significant differences in IQ measures between the groups, which were in the range typical for college students.

**Table 1:** Demographic characteristics and assessments or ratings of subjects witnessing domestic violence and unexposed controls.

	Unexposed [95% CI]	Witnessed Domestic Violence	Statistics	
Characteristics	N = 30	N = 22	(ANOVA, other)	p-value
Gender (Males/Females)	8M/22F	6M/16F	Fisher	1
Age (years)	21.6 [20.81–22.39]	21.8 [20.77–22.87]	0.122	0.86
Subject Education (years)^a^	14.28 [13.83–14.73]	13.57 [12.12–15.03]	1.18	p>0.2
**Parental Education (years)**	**16.28 [15.30–17.27]**	**14.50 [13.32–15.68]**	**5.47**	**p<0.03**
**Perceived Financial Sufficiency**	**3.63 [3.38–3.88]**	**2.77 [2.47–3.08]**	**20.25**	**p<0.00005**
**Parental Verbal Abuse Score**	**12.53 [9.86–15.20]**	**40.45 [31.68–49.23]**	**49.92**	**p<10^−8^**
Memory Assessment Scale				
Short-term memory^a,b^	111.9 [105.9–117.8]	108.8 [102.1–115.4]	0.44	p>0.5
Verbal memory^a,b^	115.6 [109.9–121.3]	113.5 [107.9–119.0]	0.28	p>0.5
Visual memory^a,b^	111.6 [107.5–115.7]	111.0 [106.3–115.7]	0.05	p>0.8
Global memory^a,b^	115.8 [111.4–120.1]	114.2 [109.6–118.8]	0.25	p>0.6
Wechsler Adult Intelligence Scale				
Verbal IQ^a,b^	127.0 [122.2–131.7]	121.8 [116.2–127.4]	2.03	p>0.1
Performance IQ^a,b^	115.9 [111.3–120.4]	114.3 [109.0–119.7]	0.19	p>0.6
Full Scale IQ^a,b^	123.6 [118.9–128.2]	120.2 [114.7–125.6]	0.90	p>0.3
Verbal Comprehension Index^a,b^	127.3 [122.8–131.8]	124.5 [119.3–129.8]	0.66	p>0.4
Perceptual Organization^a,b^	117.9 [112.7–123.1]	116.6 [110.5–122.7]	0.11	p>0.7
Working Memory Index^a,b^	116.1 [110.1–122.1]	109.7 [102.7–116.8]	1.93	p>0.1
Processing Speed Index^a,b^	112.3 [107.3–117.4]	108.2 [102.3–114.1]	1.15	p>0.2
**Anxiety^a,b^**	**4.21 [2.87–5.55]**	**8.55 [6.59–10.50]**	**15.18**	**p<0.0004**
**Depession^a,b^**	**3.03 [1.74–4.33]**	**7.41 [5.39–9.43]**	**15.43**	**p<0.0003**
**Somatization^a,b^**	**3.61 [2.14–5.07]**	**7.81 [6.09–9.53]**	**13.61**	**p<0.0007**
**Anger-Hostiity^a,b^**	**3.31 [2.33–4.29]**	**5.27 [3.88–6.67]**	**5.73**	**p<0.03**
**Limbic System Checklist-33^a,b^**	**10.57 [6.87–14.27]**	**23.18 [16.60–29.76]**	**13.6**	**p<0.0006**
**Dissociative Experience Scale^a,b^**	**4.58 [3.12–6.03]**	**14.32 [10.20–18.45]**	**26.95**	**p<10^−5^**
Drug use (days/month)^a,b^	0.36 [0.030–0.69]	0.27 [0.018–0.52]	0.16	p>0.6
**Alcohol use (drinks/month)^a,b^**	**4.84 [3.70–5.98]**	**6.76 [5.25–8.28]**	**4.37**	**p<0.05**

Adjusted for ^a^age, ^b^gender.

### Symptom Ratings and Diagnoses

Subjects in the WDV group had increased ratings of anxiety, depression, somatization, anger-hostility, dissociation and ‘limbic irritability’. Thirteen subjects in the WDV group meet DSM-IV criteria for one or more disorders. There were 9 subjects who met criteria (past or current) for major depression. Seven subjects met criteria for an anxiety disorder including four subjects with a history of PTSD. Two subjects had a past history of eating disorders, and one subject met criteria for a personality disorder. There were no differences between groups in their degree of drug use. WDV subjects consumed slightly more alcohol.

### Voxel-based Morphometry

**Figure 1:**
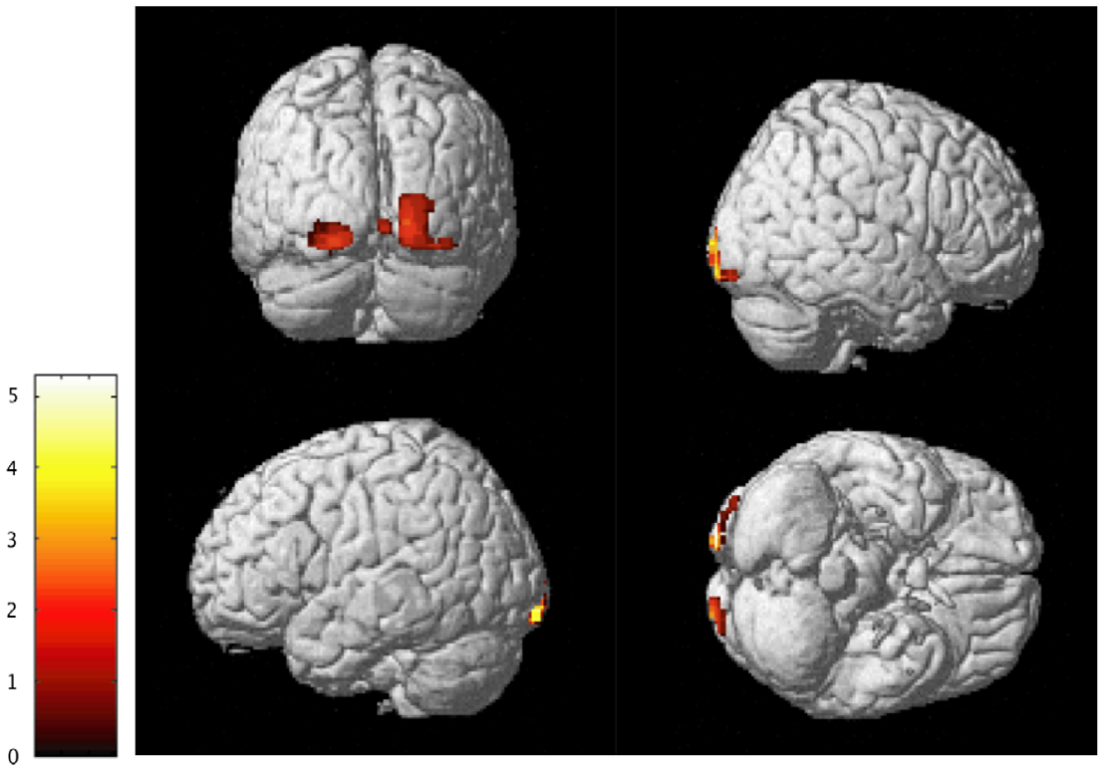
Voxel based morphometry results. Significant differences between subjects exposed to high levels of WDV and unexposed controls. Significantly decreased gray-matter densities in WDV subjects were measured in the right and left lingual gyrus (BA18). Color scale: 0–5 represent *t*-values.

The most prominent VBM finding was a 6.1% decrease in GMV in the right lingual gyrus (BA18; Talairach’s coordinates *x* = 20, *y* =  –103, *z* = 0, *Z* = 4.62, *P* = 0.029, FDR corrected peak level) (Fig. 1). The identified region consisted of a 401 voxel cluster in the right lingual gyrus ∼ inferior occipital gyrus (BA18; Talairach’s coordinates *x* = 20–36, *y* =  –103– –92, *z* =  –7–0). No other areas of decreased GMV were found with a corrected cluster or peak probability value that approached significance. Two small regions of decreased GMV were identified in the left cuneus (BA18, *x* =  –20, *y* =  –103, *z* =  –3, cluster size = 216) and right lingual gyrus (BA18, *x* = 6, *y* =  –78, *z* = 1, cluster size = 159) at an uncorrected peak level (Z = 3.94 and 3.43, respectively). There was also one small region of increased GMV in WDV subjects in the right thalamus (*x* = 14, *y* =  –23, *z* = 12, cluster size = 65) that was significant (Z = 3.47) at an uncorrected peak level.

### Cortical Thickness Measures

FreeSurfer was used to ascertain whether there were significant associations between WDV and thickness of the different portions of the visual cortex. As indicated in Table 2, the right lingual gyrus was about 6.5% thinner in WDV subjects. Further, V2 bilaterally and the left occipital pole were about 6% thinner in subjects witnessing DV.

**Table 2:** Mean and 95% confidence intervals for cortical thickness measures in the occipital region for unexposed subjects versus subjects who witnessed domestic violence.

Measures	Unexposed*	WDV Group	Group F	Group q**
right V1	1.76 [1.71–1.81]	1.68 [1.61–1.74]	2.94	p>0.2
**right V2** †	**2.16 [2.13–2.20]**	**2.03 [1.99–2.08]**	**14.65**	**p<0.007**
right MT	2.58 [2.52–2.65]	2.56 [2.48–2.65]	0.07	p>0.8
right inferior occipital gyrus and sulcus	2.77 [2.71–2.84]	2.69 [2.61–2.78]	1.53	p>0.4
right cuneus gyrus	1.87 [1.82–1.93]	1.75 [1.69–1.82]	5.38	p<0.07
right middle occipital gyrus	2.84 [2.78–2.91]	2.87 [2.79–2.94]	0.13	p>0.8
right superior occipital gyrus	2.31 [2.24–2.39]	2.40 [2.30–2.49]	1.27	p>0.4
right lateral fusiform gyrus	2.97 [2.89–3.05]	2.97 [2.87–3.07]	0.005	p>0.9
**right lingual gyrus**	**2.14 [2.09–2.19]**	**2.00 [1.94–2.07]**	**8.05**	**p<0.04**
right occipital pole	2.08 [2.02–2.13]	1.96 [1.89–2.03]	5.31	p<0.07
left V1	1.68 [1.65–1.72]	1.60 [1.55–1.64]	6.33	p<0.07
**left V2**	**2.10 [2.06–2.14]**	**1.97 [1.93–2.02]**	**13.65**	**p<0.007**
left MT	2.55 [2.50–2.61]	2.55 [2.49–2.61]	0.005	p>0.9
left inferior occipital gyrus and sulcus	2.53 [2.46–2.61]	2.45 [2.35–2.54]	1.35	p>0.4
left cuneus gyrus	1.81 [1.76–1.86]	1.79 [1.73–1.85]	0.19	p>0.8
left middle occipital gyrus	2.77 [2.71–2.83]	2.81 [2.73–2.88]	0.47	p>0.6
left superior occipital gyrus	2.29 [2.23–2.36]	2.24 [2.16–2.32]	0.69	p>0.6
left lateral fusiform gyrus	2.95 [2.88–3.01]	2.90 [2.82–2.98]	0.55	p>0.6
left lingual gyrus	1.99 [1.94–2.05]	1.87 [1.81–1.94]	5.75	p<0.07
**left occipital pole**	**2.11 [2.06–2.17]**	**1.96 [1.89–2.03]**	**8.39**	**p<0.04**

* Mean values adjusted for age, gender, parental education, preceived financial sufficiency, exposure to parental verbal abuse and mean cortical thickness.

** P values adjusted for multiple comparisons using false discovery rate (FDR) method of Benjamini and Hochberg.

† Regions highlighted in bold differed significantly between groups.

### Susceptible Versus Resilient Results

Results for the subgroup analysis comparing susceptible versus relatively resilient individuals with WDV are summarized in Table 3. Susceptible and relatively resilient subjects did not differ significantly in their extent of exposure to interparental violence. On average, resilient subjects reported 3.9±2.7 years of exposure to IP-PA and 4.8±3.9 years of exposure to IP-VA versus 4.0±4.0 and 6.2±4.6 years respectively for susceptible subjects (IP-PA: t = −0.08,df = 19.998, p>0.9; IP-VA: t = −0.79,df = 19.025, p>0.4; Welch Two Sample t-test). ANCOVA showed a significant main effect of group in the comparison between unexposed, susceptible and relatively resilient subjects in the four regions delineated in Table 2. For these regions (i.e., V2 bilaterally, right lingual gyrus, left occipital pole) it appeared to make no difference whether the subjects were susceptible or resilient, the regions were equally thin. However, this analysis also identified two regions of extriastriate visual cortex (MT/V5 bilaterally) in which there was a small but significant difference between resilient and susceptible individuals. On balance, V5/MT was about 5% thicker in resilient versus susceptible subjects exposed to WDV (both p<0.04).

**Table 3:** Mean and 95% confidence intervals for cortical thickness measures in the occipital region for unexposed subjects versus relatively resilient and susceptible individuals who witnessed domestic violence.

Measures	Unexposed*	Resilient (R)	Susceptible (S)	Group F	Group p	R vs S
right V1	1.76 [1.71–1.81]	1.69 [1.60–1.77]	1.67 [1.60–1.74]	1.55	p>0.2	p>0.8
**right V2** †	**2.16 [2.13–2.20]**	**2.03 [1.96–2.09]**	**2.04 [1.98–2.09]**	**7.22**	**p<0.002**	**p>0.9**
**right V5/MT**	**2.58 [2.52–2.64]**	**2.65 [2.55–2.76]**	**2.51 [2.42–2.60]**	**3.31**	**p<0.05**	**p<0.04**
right inferior occipital gyrus and sulcus	2.77 [2.70–2.84]	2.72 [2.61–2.84]	2.68 [2.58–2.77]	1.03	p>0.3	p>0.7
right cuneus gyrus	1.87 [1.82–1.93]	1.75 [1.66–1.84]	1.76 [1.68–1.83]	2.64	p<0.1	p>0.9
right middle occipital gyrus	2.85 [2.78–2.91]	2.86 [2.75–2.96]	2.87 [2.78–2.96]	0.10	p>0.9	p>0.9
right superior occipital gyrus	2.31 [2.24–2.39]	2.46 [2.34–2.59]	2.35 [2.25–2.46]	1.96	p>.1	p>0.2
right lateral fusiform gyrus	2.97 [2.89–3.05]	2.99 [2.85–3.13]	2.95 [2.84–3.07]	0.12	p>0.8	p>0.8
**right lingual gyrus**	**2.14 [2.09–2.19]**	**2.00 [1.91–2.08]**	**2.01 [1.93–2.08]**	**3.96**	**p<0.03**	**p>0.9**
right occipital pole	2.08 [2.02–2.13]	1.95 [1.86–2.04]	1.96 [1.88–2.04]	2.62	p<0.1	p>0.9
left V1	1.69 [1.65–1.72]	1.59 [1.53–1.65]	1.60 [1.55–1.66]	3.16	p<0.06	p>0.9
**left V2**	**2.10 [2.06–2.14]**	**1.99 [1.93–2.05]**	**1.96 [1.91–2.02]**	**7.03**	**p<0.003**	**p>0.6**
**left V5/MT**	**2.55 [2.50–2.60]**	**2.62 [2.54–2.70]**	**2.51 [2.44–2.58]**	**3.22**	**p<0.05**	**p<0.05**
left inferior occipital gyrus and sulcus	2.53 [2.46–2.60]	2.52 [2.39–2.64]	2.40 [2.30–2.51]	2.12	p>0.1	p>0.2
left cuneus gyrus	1.81 [1.76–1.86]	1.79 [1.70–1.87]	1.79 [1.72–1.86]	0.09	p>0.9	p>0.9
left middle occipital gyrus	2.77 [2.71–2.83]	2.83 [2.73–2.93]	2.79 [2.71–2.88]	0.47	p>0.6	p>0.7
left superior occipital gyrus	2.29 [2.23–2.35]	2.32 [2.21–2.43]	2.19 [2.10–2.28]	2.90	p<0.07	p<0.08
left lateral fusiform gyrus	2.95 [2.88–3.01]	2.94 [2.83–3.05]	2.88 [2.79–2.97]	0.85	p>0.4	p>0.5
left lingual gyrus	1.99 [1.94–2.05]	1.88 [1.79–1.97]	1.87 [1.79–1.95]	2.85	p<0.07	p>0.9
**left occipital pole**	**2.11 [2.06–2.17]**	**1.92 [1.83–2.02]**	**1.98 [1.90–2.06]**	**4.93**	**p<0.02**	**p>0.5**

* Mean values adjusted for age, gender, parental education, preceived financial sufficiency, exposure to parental verbal abuse and mean cortical thickness.

† Regions highlighted in bold differed significantly between groups.

### Witnessing Interparental Verbal Versus Physical Abuse Results on GMV

**Figure 2:**
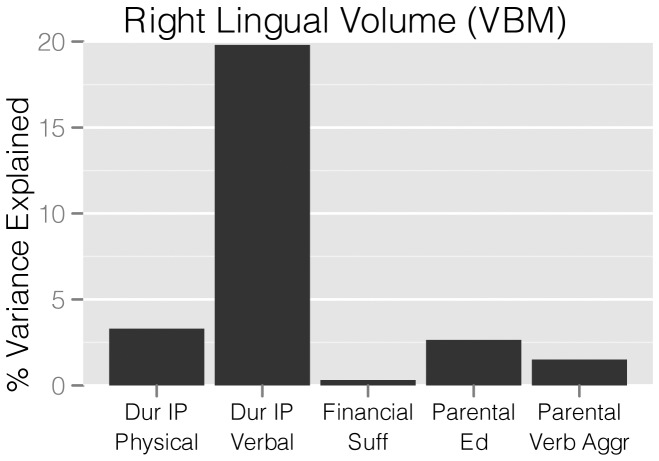
Relative importance – linear regression. Linear regression with variance decomposition indicating the percent of variance in adjusted gray matter volume from peak VBM cluster accounted for by regressors. Dur IP Physical – duration of witnessing interparental physical aggression (years), Dur IP Verbal – duration of witnessing interparental verbal aggression without physical violence (years), Financial Suf – perceived financial sufficiency during childhood, Parental Ed – average parental education (years), Parental Verb Aggr – average parental verbal aggression score.

The strength of the statistical association between duration of exposure to IP-VA versus IP-PA and GMV of the right lingual gyrus is illustrated in Figure 2. Duration of exposure to IP-VA accounted for 19.8% of the variance in GMV of this region. In contrast, duration of exposure to IP-PA accounted for only 3.2% of the variance.

### Witnessing Interparental Verbal Versus Physical Abuse Results on Thickness

**Figure 3:**
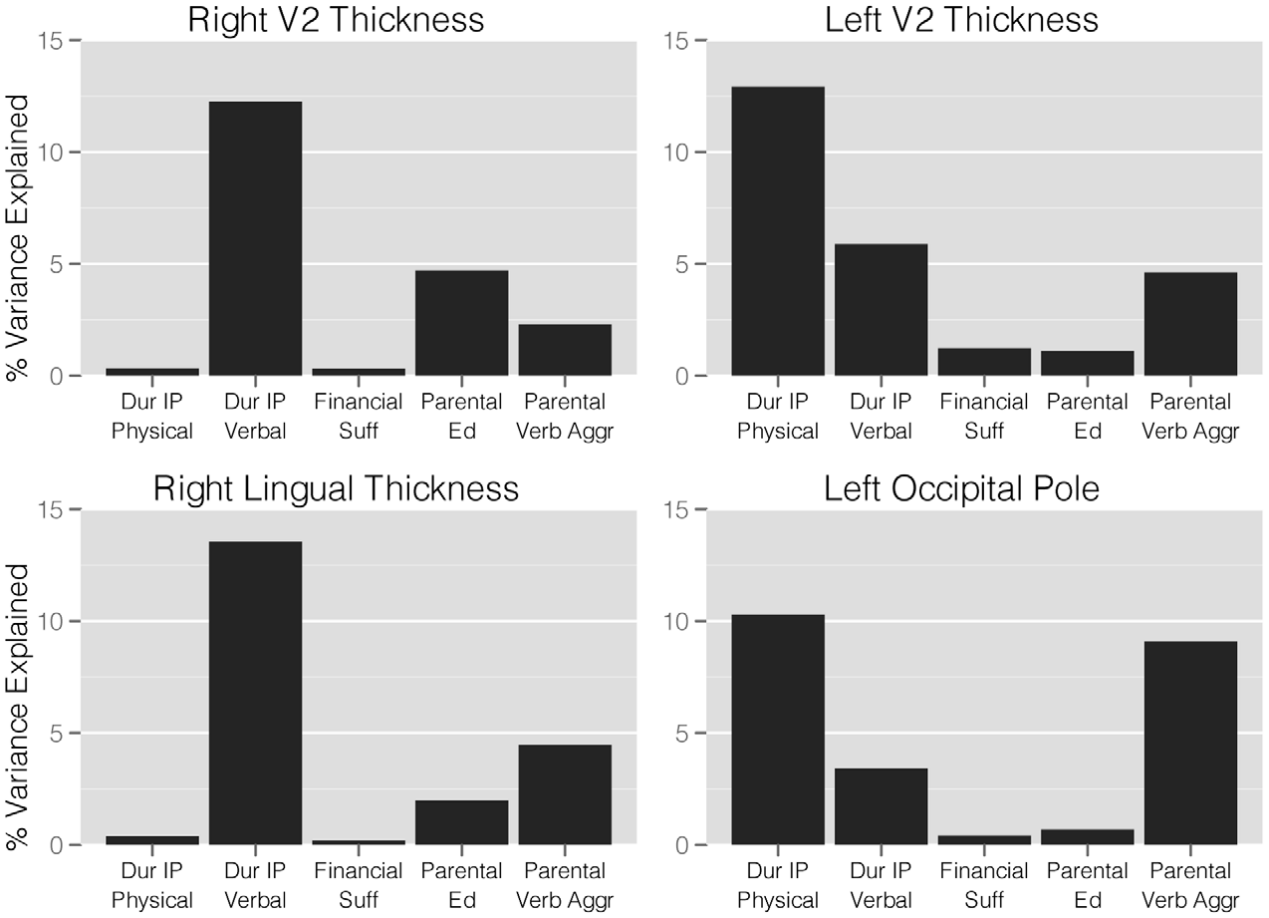
Relative importance – linear regression. Linear regression with variance decomposition indicating the percent of variance in adjusted regional cortical thickness measures from FreeSurfer accounted for by regressors. Dur IP Physical – duration of witnessing interparental physical aggression (years), Dur IP Verbal – duration of witnessing interparental verbal aggression without physical violence (years), Financial Suf – perceived financial sufficiency during childhood, Parental Ed – average parental education (years), Parental Verb Aggr – average parental verbal aggression score.

Figure 3 illustrates the results of the same analyses applied to the FreeSurfer thickness measures. Duration of exposure to IP-VA accounted for a much greater share of the variance then exposure to IP-PA in thickness measures for right V2 and right lingual gyrus. In contrast, duration of exposure to IP-PA accounted for a greater share of the variance then exposure to IP-VA in thickness measures for left V2 and left occipital pole.

### Sensitive Period Results

**Figure 4:**
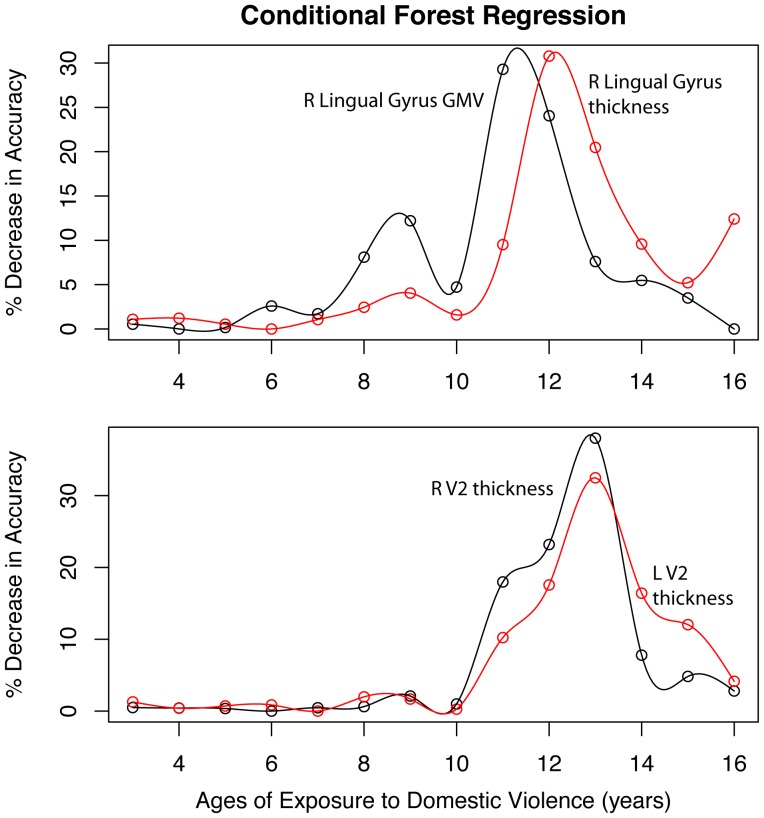
Relative importance – conditional forest regression. Relative importance of exposure to interparental aggression during specific years of childhood on adjusted measures of gray matter volume or thickness in right lingual gyrus and V2 bilaterally. Results based on random forest regression with conditional trees indicating the percent decrease in accuracy of the fit following permutation of each exposure age.

Results of conditional forest regression analyses are illustrated in Figure 4. Overall, exposure age accounted for 17% (p = 0.002) and 13% (p<0.008) of the variance in right lingual gyrus GMV and thickness, respectively. Similarly, exposure age accounted for 14% (p<0.006) and 11% (p<0.02) of the variance in thickness of right and left V2. The importance of exposure at each age was not however uniform. The most important predictors of right lingual gyrus GMV and thickness were exposure at 11–12, and 12–13 years of age, respectively. Similarly, exposures at 12–13 years were key predictors of left and right V2 thickness. Peak sensitivity occurred at age 11 for right lingual gyrus GMV, age 12 for right lingual gyrus thickness and age 13 for V2 thickness bilaterally.

### Right Lingual Gyrus GMV and Symptom Ratings

As indicated in Table 4, there were significant associations between GMV in right lingual gyrus (BA18: Talairach’s coordinates x = 6, y =  –80, z =  –4) and symptoms of dissociation and limbic irritability across the entire subject pool. These statistical associations were most discernible in the LSCL-33 subscales measuring somatization and dissociation. Partial correlations between GMV in right lingual gyrus and LSCL-33 were discernible in the WDV group but not in the control group.

**Table 4:** Partial correlations between gray matter volume in right lingual gyrus and symptom ratings, controlling for age and gender.

	All Subjects		WDV Group		Healthy Controls	
Ratings	Correlation	P value	Correlation	P value	Correlation	P value
Dissociation (DES)	**−0.31**	**p<0.03**	−0.32	p<0.16	0.32	p<0.09
Limbic Irritability (LSCL-33)	**−0.36**	**p<0.009**	**−0.49**	**p<0.02**	0.17	p>0.3
LSCL - Somatization	**−0.39**	**p<0.004**	**−0.46**	**p<0.03**	0.04	p>0.8
LSCL - Hallucinations	−0.26	p<0.06	−0.37	p<0.10	0.11	p>0.5
LSCL - Automatisms	−0.25	p<0.08	−0.32	p<0.15	0.28	p<0.14
LSCL - Dissociation	**−0.35**	**p = 0.01**	**−0.50**	**p<0.02**	0.25	p<0.2
Anxiety (SQ)	−0.26	p = 0.06	−0.30	p<0.2	0.12	p>0.5
Depression (SQ)	−0.23	p = 0.10	−0.27	p>0.2	0.26	p<0.18
Somatization (SQ)	−0.12	p>0.4	−0.07	p>0.7	0.20	p>0.3
Hostility (SQ)	−0.10	p>0.5	−0.15	p>0.5	0.22	p>0.2

Abbreviations: DES - Dissociative Experience Scale, LSCL-33 - Limbic System Checklist - 33, SQ - Symptom Questionnaire, WDV - Witnessing Domestic Violence.

Values in bold are statistically significant.

## Discussion

VBM identified a significant association between exposure to WDV and reduced GMV in the right lingual gyrus. Analysis using cortical surface parcellation also provided evidence for statistically significant differences in the thickness of the right lingual gyrus, left occipital pole and V2 bilaterally.

This right lingual gyrus plays a critical role in global aspects of figure recognition [39] and object naming [40]. It is characterized anatomically as a unimodal sensory area [41], though its response to visual stimuli is modulated by other types of sensory information through cortical back projections [41]. It may also be an important substrate for dreaming [42] and colors [43], and is a brain region that consistently shows a reduction in cerebral blood flow after sleep deprivation or disruption [44], [45]. Sleep disruption caused by WDV may diminish activity and blood flow to this region, and consequently alter its developmental trajectory. V2 is a component of early visual cortex that appears to be essential for conscious visual awareness [46]. Similarly, V5/MT plays a critical role in the conscious perception of visual movement [47]. Recent studies suggest that visual awareness depends on feedback from regions of extrastriate visual cortex (e.g., V2, V5/MT) back to V1 [46], [47]. The occipital pole is the most posterior portion of the occipital cortex and is the major component of V1.

These results are similar to findings previously reported in subjects exposed to CSA [48]. Those individuals had reduced GMV in V1 and V2 bilaterally, with most prominent differences appearing in right lingual gyrus and left fusiform gyrus using FreeSurfer [48]. These observations also fit with results of our previous WDV DTI analysis, which showed reduced FA in the left ILF connecting occipital cortex and limbic regions [8]. Reduced occipital GMV was also found by Fennema-Notestine et al., [49] to be associated with a prior history of childhood abuse. Further, diminished activation of right visual association areas was reported in a PET study of women with CSA-related PTSD [50]. Hence, different studies using an array of techniques provide complementary evidence for a potential association between exposure to certain forms of childhood abuse and structure or function of the visual cortex.

Interestingly, we observed a very different pattern of results in a sample of young adults exposed to high levels of parental verbal abuse but not WDV, CSA or PA. VBM revealed an increase in GMV in left superior temporal gyrus (auditory cortex) of verbally-abused subjects [11], and DTI analyses showed reduced FA in the arcuate fasciculus interconnecting Wernicke's and Broca’s areas [51]. Together these findings suggest that sensory systems that process and interpret the adverse sensory inputs may be modified by the exposure.

The present findings also revealed a potential sensitive period, between 11–13 years of age, when exposure to WDV exerted maximal effects on GMV or thickness. This fits with our prior observation of a sensitive period between 7–13 years for WDV and myelination of the ILF [8], and with our earlier observation that CSA was associated with a reduction in occipital cortex GMV if it occurred prior to age 12 (in an all female sample) but not after [48]. Research has shown that plasticity of the visual cortex abates following puberty [52], and that human perceptual development remains vulnerable to damage from adverse visual experience until 10 to 13 years of age [53]. The present findings suggest that visual cortex may be particularly vulnerable to WDV during the peripubertal period.

The present findings also expand on our prior observation that visually witnessing IP-VA was associated with greater statistical effects on FA in the ILF then witnessing IP-PA [8]. GMV and thickness of the right lingual gyrus and right V2 in the current study were strongly influenced by duration of exposure to IP-VA but not IP-PA. On the other hand, thickness in left V2 and left occipital pole appeared to be more strongly influenced by duration of exposure to IP-PA then IP-VA. This is an intriguing observation of potential hemispheric differences that, if replicable, suggests a considerable degree of complexity in the association between type of exposure and morphometric measures.

Differences between WDV and controls in these regions were apparent in both relatively resilient and susceptible individuals. We had previously reported that reduced GMV in visual cortex was observable in both susceptible and resilient subjects with CSA [48], and that reduced FA in the ILF in WDV subjects was not mediated by presence or severity of psychiatric symptoms [8]. The presence of discernible (and potentially equivalent) neurobiological abnormalities in psychiatrically-resilient versus susceptible individuals with maltreatment histories appears to be an emerging trend. For example, we recently reported a strong association between severity of exposure to maltreatment and hippocampal subfield volume in dentate gyrus and CA3 that was unrelated to history or severity of depression or PTSD [13]. Similarly, Dannlowski et al. [54] reported reduced hippocampal volume, and increased anygdala reactivity in maltreated subjects without psychopathology. Additional evidence for amygdala hyperreactivity in maltreated individuals without psychopathology has also been reported by McCrory et al. [55] and van Harmelen et al. [56]. This has led to the speculation that these neurobiological correlates of exposure may be more of a risk factor for psychopathology than a consequence.

Interestingly, though reductions in right lingual GMV was observed in both susceptible and resilient subjects, there were significant associations between GMV in this region and self-report ratings of dissociation and limbic irritability. The most strongly associated subscale of the LSCL-33 in the WDV group consisted of items such as “The sensations that events, conversations, or a place was strangely familiar, as if you had experienced or dreamed the situation before” and “The sensation that your mind has left your body, or that you are watching yourself as a detached observer”. While resilient subjects exposed to DV had ratings of depression and anxiety that were no greater than controls (and substantially lower than in susceptible individuals), they had dissociative experience scores nearly equal to susceptible subjects and much greater than controls. Hence, although resilient subjects in the WDV group did not experience the most common psychiatric consequences of exposure (depression and anxiety) they did experience heightened levels of dissociation. Alterations in the development of the right lingual gyrus or the ILF [8] (which interconnects lingual gyrus with hippocampus) may play a role in the generation of this phenomenon. This may result from: (1) a problem in the cross-modal influence of other sensory systems on visual perception [41]; (2) impaired integration of visual perceptions and hippocampal contextual memories through the ILF; or (3) intrusion of dream-like visual imagery (associated with right lingual gyrus [42]) into wake time.

The present study is relatively unique in its focus on the potential consequences of exposure to a specific type of abuse. This approach has been useful in revealing similarities and differences between the neurobiological correlates of exposure to childhood sexual abuse [48], [57], parental verbal abuse [11], [51], WDV [8] and harsh corporal punishment [12], [58]. This strength is also a limitation as many abused individuals, particularly those involved in the mental health system, experienced multiple forms of maltreatment. Hamby et al. [59] reported in a nationally representative sample, that 56.6% of youth witnessing interparental violence would, over the course of their lifetime, experience other forms of maltreatment. Hence, our findings are more applicable to the remaining 43%, who experience WDV as their sole form of maltreatment.

Studies of individuals exposed to single types of maltreatment have primarily identified differences in sensory regions or pathways [8], [11], [48], [51]. This observation stands in contrast to findings from other studies, including our own, that combine subjects exposed to one or more types of abuse into a single group. Those studies have predominantly identified alterations in corpus callosum, hippocampus and frontal cortex [13], [54], [60], [61], [62], [63], [64], [65], [66], [67], [68], [69], [70], [71]. It may be the case that chronic exposure to a specific type of adversity primarily affects the development of sensory systems that process or convey the adverse sensory input. In contrast, neurobiological response (or adaptation) to multiple forms of adversity may occur predominantly at limbic or frontal cortical levels and affect interhemispheric communication. This view is compatible with the observation that risk for psychopathology is much greater in individuals exposed to multiple types of maltreatment then single forms [17], [72].

The main limitation of this study is the relatively small sample size. A large, initial sample of 18- to 25-year-olds was surveyed to identify a healthy sample of subjects in the community, as opposed to psychiatric sources, who were exposed only to WDV and to no other forms of trauma or early adversity. Exposure to high levels of WDV but to no other forms of abuse is a relatively common occurrence, reported by about 4% of subjects in this age range [17]. Our findings should generalize to subjects experiencing WDV or WDV plus parental verbal abuse, but no other forms of abuse, as we selected subjects without regard to psychopathology (except substance abuse). It remains to be seen if the same findings emerge in subjects exposed to WDV plus sexual or physical abuse.

Although this study revealed a significant association between a self-reported history of WDV and decreased GMV or thickness in visual cortex, it should be emphasized that the finding is correlational and does not prove that WDV caused the decrease. Prospective longitudinal studies are required to establish a causal relationship. Nevertheless, these findings are consistent with a causal relationship and suggest that exposure to WDV may act as a traumatic stressor to alter the development of the visual cortex. If so, these results underscore efforts to prevent children from exposure to acts of domestic violence and other forms of abuse or neglect.

## References

[1] McDonald R, Jouriles EN, Ramisetty-Mikler S, Caetano R, Green CE (2006) Estimating the number of American children living in partner-violent families. J Fam Psychol 20: 137–142. 16569098 10.1037/0893-3200.20.1.137

[2] Fantuzzo J, Boruch R, Beriama A, Atkins M, Marcus S (1997) Domestic violence and children: prevalence and risk in five major U.S. cities. J Am Acad Child Adolesc Psychiatry 36: 116–122. 9000789 10.1097/00004583-199701000-00025

[3] Nicodimos S, Gelaye BS, Williams MA, Berhane Y (2009) Associations between witnessing parental violence and experiencing symptoms of depression among college students. East Afr J Public Health 6: 184–190. 20000027 10.4314/eajph.v6i2.51764 PMC3003665

[4] Abrahams N, Jewkes R (2005) Effects of South African men's having witnessed abuse of their mothers during childhood on their levels of violence in adulthood. Am J Public Health 95: 1811–1816. 16131646 10.2105/AJPH.2003.035006 PMC1449441

[5] Casiano H, Mota N, Afifi TO, Enns MW, Sareen J (2009) Childhood maltreatment and threats with weapons. J Nerv Ment Dis 197: 856–861. 19996725 10.1097/NMD.0b013e3181be9c55

[6] Luthra R, Abramovitz R, Greenberg R, Schoor A, Newcorn J, et al (2009) Relationship between type of trauma exposure and posttraumatic stress disorder among urban children and adolescents. J Interpers Violence 24: 1919–1927. 18945918 10.1177/0886260508325494

[7] Silva RR, Alpert M, Munoz DM, Singh S, Matzner F, et al (2000) Stress and vulnerability to posttraumatic stress disorder in children and adolescents. Am J Psychiatry 157: 1229–1235. 10910784 10.1176/appi.ajp.157.8.1229

[8] Choi J, Jeong B, Polcari A, Rohan ML, Teicher MH (2012) Reduced fractional anisotropy in the visual limbic pathway of young adults witnessing domestic violence in childhood. Neuroimage 59: 1071–1079. 21985907 10.1016/j.neuroimage.2011.09.033 PMC3236680

[9] Dale AM, Fischl B, Sereno MI (1999) Cortical surface-based analysis. I. Segmentation and surface reconstruction. Neuroimage 9: 179–194. 9931268 10.1006/nimg.1998.0395

[10] Fischl B, Sereno MI, Dale AM (1999) Cortical surface-based analysis. II: Inflation, flattening, and a surface-based coordinate system. Neuroimage 9: 195–207. 9931269 10.1006/nimg.1998.0396

[11] Tomoda A, Sheu YS, Rabi K, Suzuki H, Navalta CP, et al (2011) Exposure to parental verbal abuse is associated with increased gray matter volume in superior temporal gyrus. Neuroimage 54 Suppl 1S280–286. 20483374 10.1016/j.neuroimage.2010.05.027 PMC2950228

[12] Tomoda A, Suzuki H, Rabi K, Sheu YS, Polcari A, et al (2009) Reduced prefrontal cortical gray matter volume in young adults exposed to harsh corporal punishment. Neuroimage 47 Suppl 2T66–71. 19285558 10.1016/j.neuroimage.2009.03.005 PMC2896871

[13] Teicher MH, Anderson CM, Polcari A (2012) Childhood maltreatment is associated with reduced volume in the hippocampal subfields CA3, dentate gyrus, and subiculum. Proc Natl Acad Sci U S A 109: E563–572. 22331913 10.1073/pnas.1115396109 PMC3295326

[14] First MB, Spitzer RL, Gibbon M, Williams JBW (1997) Structured clinical interview for DSM-IV axis I disorders - clinician version (SCID-CV). Washington, DC: American Psychiatric Press.

[15] Herman JL, Perry JC, van der Kolk BA (1989) Traumatic Antecedents Interview. Boston: The Trauma Center.

[16] Roy CA, Perry JC (2004) Instruments for the assessment of childhood trauma in adults. J Nerv Ment Dis 192: 343–351. 15126888 10.1097/01.nmd.0000126701.23121.fa

[17] Teicher MH, Samson JA, Polcari A, McGreenery CE (2006) Sticks, stones, and hurtful words: relative effects of various forms of childhood maltreatment. Am J Psychiatry 163: 993–1000. 16741199 10.1176/ajp.2006.163.6.993

[18] Kellner R (1987) A symptom questionnaire. Journal of Clinical Psychiatry 48: 268–273. 3597327

[19] Bernstein DP, Fink L (1998) Childhood Trauma Questionnaire Manual. San Antonio, TX: The Psychological Corporation.

[20] Teicher MH, Glod CA, Surrey J, Swett C Jr (1993) Early childhood abuse and limbic system ratings in adult psychiatric outpatients. J Neuropsychiatry Clin Neurosci 5: 301–306. 8369640 10.1176/jnp.5.3.301

[21] Good CD, Johnsrude IS, Ashburner J, Henson RN, Friston KJ, et al (2001) A voxel-based morphometric study of ageing in 465 normal adult human brains. Neuroimage 14: 21–36. 11525331 10.1006/nimg.2001.0786

[22] Good CD, Johnsrude I, Ashburner J, Henson RN, Friston KJ, et al (2001) Cerebral asymmetry and the effects of sex and handedness on brain structure: a voxel-based morphometric analysis of 465 normal adult human brains. Neuroimage 14: 685–700. 11506541 10.1006/nimg.2001.0857

[23] Ashburner J, Friston KJ (2000) Voxel-based morphometry–the methods. Neuroimage 11: 805–821. 10860804 10.1006/nimg.2000.0582

[24] Ashburner J, Friston KJ (2005) Unified segmentation. Neuroimage 26: 839–851. 15955494 10.1016/j.neuroimage.2005.02.018

[25] Hayasaka S, Phan KL, Liberzon I, Worsley KJ, Nichols TE (2004) Nonstationary cluster-size inference with random field and permutation methods. Neuroimage 22: 676–687. 15193596 10.1016/j.neuroimage.2004.01.041

[26] Moorhead TW, Job DE, Spencer MD, Whalley HC, Johnstone EC, et al (2005) Empirical comparison of maximal voxel and non-isotropic adjusted cluster extent results in a voxel-based morphometry study of comorbid learning disability with schizophrenia. Neuroimage 28: 544–552. 16085427 10.1016/j.neuroimage.2005.04.045

[27] Friston KJ, Holmes A, Poline JB, Price CJ, Frith CD (1996) Detecting activations in PET and fMRI: levels of inference and power. Neuroimage 4: 223–235. 9345513 10.1006/nimg.1996.0074

[28] Worsley KJ, Andermann M, Koulis T, MacDonald D, Evans AC (1999) Detecting changes in nonisotropic images. Hum Brain Mapp 8: 98–101. 10524599 10.1002/(SICI)1097-0193(1999)8:2/3<98::AID-HBM5>3.0.CO;2-F PMC6873343

[29] Bookstein FL (2001) “Voxel-based morphometry” should not be used with imperfectly registered images. Neuroimage 14: 1454–1462. 11707101 10.1006/nimg.2001.0770

[30] Fischl B, Liu A, Dale AM (2001) Automated manifold surgery: constructing geometrically accurate and topologically correct models of the human cerebral cortex. IEEE Trans Med Imaging 20: 70–80. 11293693 10.1109/42.906426

[31] Hochberg Y, Benjamini Y (1990) More powerful procedures for multiple significance testing. Stat Med 9: 811–818. 2218183 10.1002/sim.4780090710

[32] R Development Core Team (2010) R: A Language and Environment for Statistical Computing. Vienna, Austria: R Foundation for Statistical Computing.

[33] Lindeman RH, Merenda PF, Gold RZ (1980) Introduction to Bivariate and Multivariate Analysis. Glenview, IL: Scott, Foresman.

[34] Grömping U (2007) Estimators of relative importance in linear regression based on variance decomposition. The American Statistician 61: 139–147.

[35] Strobl C, Boulesteix AL, Zeileis A, Hothorn T (2007) Bias in random forest variable importance measures: illustrations, sources and a solution. BMC Bioinformatics 8: 25. 17254353 10.1186/1471-2105-8-25 PMC1796903

[36] Breiman L (2001) Random Forests. Machine Learning 45: 5–32.

[37] Cutler DR, Edwards TC, Beard KH, Cutler A, Hess KT, et al (2007) Random forests for classification in ecology. Ecology 88: 2783–2792. 18051647 10.1890/07-0539.1

[38] Russo R (2003) Statistics for the Behavioral Sciences: An Introduction: Psychology Press. 192–193 p.

[39] Fink GR, Halligan PW, Marshall JC, Frith CD, Frackowiak RS, et al (1996) Where in the brain does visual attention select the forest and the trees? Nature 382: 626–628. 8757132 10.1038/382626a0

[40] Kiyosawa M, Inoue C, Kawasaki T, Tokoro T, Ishii K, et al (1996) Functional neuroanatomy of visual object naming: a PET study. Graefes Arch Clin Exp Ophthalmol 234: 110–115. 8720681 10.1007/BF00695250

[41] Macaluso E, Frith CD, Driver J (2000) Modulation of human visual cortex by crossmodal spatial attention. Science 289: 1206–1208. 10947990 10.1126/science.289.5482.1206

[42] Bischof M, Bassetti CL (2004) Total dream loss: a distinct neuropsychological dysfunction after bilateral PCA stroke. Ann Neurol 56: 583–586. 15389890 10.1002/ana.20246

[43] Howard RJ, ffytche DH, Barnes J, McKeefry D, Ha Y, et al (1998) The functional anatomy of imagining and perceiving colour. Neuroreport 9: 1019–1023. 9601660 10.1097/00001756-199804200-00012

[44] Joo EY, Tae WS, Han SJ, Cho JW, Hong SB (2007) Reduced cerebral blood flow during wakefulness in obstructive sleep apnea-hypopnea syndrome. Sleep 30: 1515–1520. 18041484 10.1093/sleep/30.11.1515 PMC2082095

[45] Joo EY, Seo DW, Tae WS, Hong SB (2008) Effect of modafinil on cerebral blood flow in narcolepsy patients. Sleep 31: 868–873. 18548832 10.1093/sleep/31.6.868 PMC2442416

[46] Salminen-Vaparanta N, Koivisto M, Noreika V, Vanni S, Revonsuo A (2012) Neuronavigated transcranial magnetic stimulation suggests that area V2 is necessary for visual awareness. Neuropsychologia 50: 1621–1627. 22465860 10.1016/j.neuropsychologia.2012.03.015

[47] Silvanto J, Lavie N, Walsh V (2005) Double dissociation of V1 and V5/MT activity in visual awareness. Cereb Cortex 15: 1736–1741. 15703247 10.1093/cercor/bhi050

[48] Tomoda A, Navalta CP, Polcari A, Sadato N, Teicher MH (2009) Childhood sexual abuse is associated with reduced gray matter volume in visual cortex of young women. Biol Psychiatry 66: 642–648. 19560122 10.1016/j.biopsych.2009.04.021 PMC4277202

[49] Fennema-Notestine C, Stein MB, Kennedy CM, Archibald SL, Jernigan TL (2002) Brain morphometry in female victims of intimate partner violence with and without posttraumatic stress disorder. Biol Psychiatry 52: 1089–1101. 12460692 10.1016/s0006-3223(02)01413-0

[50] Bremner JD, Vermetten E, Vythilingam M, Afzal N, Schmahl C, et al (2004) Neural correlates of the classic color and emotional stroop in women with abuse-related posttraumatic stress disorder. Biol Psychiatry 55: 612–620. 15013830 10.1016/j.biopsych.2003.10.001

[51] Choi J, Jeong B, Rohan ML, Polcari AM, Teicher MH (2009) Preliminary evidence for white matter tract abnormalities in young adults exposed to parental verbal abuse. Biol Psychiatry 65: 227–234. 18692174 10.1016/j.biopsych.2008.06.022 PMC2652864

[52] Hubel DH, Wiesel TN (1998) Early exploration of the visual cortex. Neuron 20: 401–412. 9539118 10.1016/s0896-6273(00)80984-8

[53] Lewis TL, Maurer D (2005) Multiple sensitive periods in human visual development: evidence from visually deprived children. Dev Psychobiol 46: 163–183. 15772974 10.1002/dev.20055

[54] Dannlowski U, Stuhrmann A, Beutelmann V, Zwanzger P, Lenzen T, et al (2012) Limbic scars: long-term consequences of childhood maltreatment revealed by functional and structural magnetic resonance imaging. Biol Psychiatry 71: 286–293. 22112927 10.1016/j.biopsych.2011.10.021

[55] McCrory EJ, De Brito SA, Sebastian CL, Mechelli A, Bird G, et al (2011) Heightened neural reactivity to threat in child victims of family violence. Current Biology 21: R947–R948. 22153160 10.1016/j.cub.2011.10.015

[56] van Harmelen AL, van Tol MJ, Demenescu LR, van der Wee NJ, Veltman DJ, et al.. (2012) Enhanced amygdala reactivity to emotional faces in adults reporting childhood emotional maltreatment. Soc Cogn Affect Neurosci. 10.1093/scan/nss007 PMC3624946 22258799

[57] Andersen SL, Tomoda A, Vincow ES, Valente E, Polcari A, et al (2008) Preliminary evidence for sensitive periods in the effect of childhood sexual abuse on regional brain development. J Neuropsychiatry Clin Neurosci 20: 292–301. 18806232 10.1176/appi.neuropsych.20.3.292 PMC4270804

[58] Sheu YS, Polcari A, Anderson CM, Teicher MH (2010) Harsh corporal punishment is associated with increased T2 relaxation time in dopamine-rich regions. Neuroimage 53: 412–419. 20600981 10.1016/j.neuroimage.2010.06.043 PMC3854930

[59] Hamby S, Finkelhor D, Turner H, Ormrod R (2010) The overlap of witnessing partner violence with child maltreatment and other victimizations in a nationally representative survey of youth. Child Abuse Negl 34: 734–741. 20850182 10.1016/j.chiabu.2010.03.001

[60] Stein MB, Koverola C, Hanna C, Torchia MG, McClarty B (1997) Hippocampal volume in women victimized by childhood sexual abuse. Psychol Med 27: 951–959. 9234472 10.1017/s0033291797005242

[61] Bremner JD, Randall P, Vermetten E, Staib L, Bronen RA, et al (1997) Magnetic resonance imaging-based measurement of hippocampal volume in posttraumatic stress disorder related to childhood physical and sexual abuse–a preliminary report. Biol Psychiatry 41: 23–32. 8988792 10.1016/s0006-3223(96)00162-x PMC3229101

[62] Vermetten E, Schmahl C, Lindner S, Loewenstein RJ, Bremner JD (2006) Hippocampal and amygdalar volumes in dissociative identity disorder. Am J Psychiatry 163: 630–636. 16585437 10.1176/appi.ajp.163.4.630 PMC3233754

[63] Vythilingam M, Heim C, Newport J, Miller AH, Anderson E, et al (2002) Childhood trauma associated with smaller hippocampal volume in women with major depression. Am J Psychiatry 159: 2072–2080. 12450959 10.1176/appi.ajp.159.12.2072 PMC3230324

[64] De Bellis MD, Keshavan MS, Clark DB, et al (1999) A.E. Bennett Research Award. Developmental traumatology. Part II: Brain development. Biol Psychiatry 45: 1271–1284. 10349033 10.1016/s0006-3223(99)00045-1

[65] De Bellis MD, Keshavan MS, Frustaci K, Shifflett H, Iyengar S, et al (2002) Superior temporal gyrus volumes in maltreated children and adolescents with PTSD. Biol Psychiatry 51: 544–552. 11950456 10.1016/s0006-3223(01)01374-9

[66] Teicher MH, Dumont NL, Ito Y, Vaituzis C, Giedd JN, et al (2004) Childhood neglect is associated with reduced corpus callosum area. Biol Psychiatry 56: 80–85. 15231439 10.1016/j.biopsych.2004.03.016

[67] Jackowski AP, Douglas-Palumberi H, Jackowski M, Win L, Schultz RT, et al (2008) Corpus callosum in maltreated children with posttraumatic stress disorder: a diffusion tensor imaging study. Psychiatry Res 162: 256–261. 18296031 10.1016/j.pscychresns.2007.08.006 PMC3771642

[68] Carrion VG, Weems CF, Reiss AL (2007) Stress predicts brain changes in children: a pilot longitudinal study on youth stress, posttraumatic stress disorder, and the hippocampus. Pediatrics 119: 509–516. 17332204 10.1542/peds.2006-2028

[69] Richert KA, Carrion VG, Karchemskiy A, Reiss AL (2006) Regional differences of the prefrontal cortex in pediatric PTSD: an MRI study. Depress Anxiety 23: 17–25. 16247760 10.1002/da.20131

[70] Teicher MH, Samson JA, Sheu YS, Polcari A, McGreenery CE (2010) Hurtful words: association of exposure to peer verbal abuse with elevated psychiatric symptom scores and corpus callosum abnormalities. Am J Psychiatry 167: 1464–1471. 20634370 10.1176/appi.ajp.2010.10010030 PMC3246683

[71] Edmiston EE, Wang F, Mazure CM, Guiney J, Sinha R, et al (2011) Corticostriatal-Limbic Gray Matter Morphology in Adolescents With Self-reported Exposure to Childhood Maltreatment. Arch Pediatr Adolesc Med 165: 1069–1077. 22147775 10.1001/archpediatrics.2011.565 PMC3607102

[72] Anda RF, Felitti VJ, Bremner JD, Walker JD, Whitfield C, et al (2006) The enduring effects of abuse and related adverse experiences in childhood: A convergence of evidence from neurobiology and epidemiology. Eur Arch Psychiatry Clin Neurosci 256: 174–186. 16311898 10.1007/s00406-005-0624-4 PMC3232061

